# Influence of Electrode Structure on Performance of Laser Direct Writing Cu-PI Flexible Humidity Sensor

**DOI:** 10.3390/mi13070992

**Published:** 2022-06-24

**Authors:** Jipeng Zhao, Zixiao Yu, Zhenyue Tu, Hongxia Bian

**Affiliations:** 1 Gansu Province Special Equipment Inspection and Testing Institute, Lanzhou 730050, China; zhaojipeng2001@163.com; 2 College of Science, Gansu Agricultural University, Lanzhou 730070, China; 17751519361@163.com

**Keywords:** humidity sensor, electrode, laser direct writing, CuO

## Abstract

Electrode structure is an essential factor affecting the performance of flexible humidity sensors. In this study, Cu and Cu2 + 1O electrodes were printed by the one-step method using laser direct writing technology to reduce the nano–CuO ink on flexible substrate PI and to be used for a humidity sensor. The resistance of the humidity sensors with nine various electrode structures was measured under the relative humidity (RH) of 16–78%. It was observed that all sensors showed good humidity sensing characteristics, and the sensitivity of the copper-based humidity sensor was not affected by the electrode structure under low humidity conditions but was significant under high humidity conditions. The sensor with the length of 1960 μm and the width of 120 μm shows the lowest sensitivity of 180.2 KΩ/%RH under 35% RH, and the sensor with the length of 2430 μm and the width of 180 μm shows the highest sensitivity of 1744 kΩ/%RH under 65% RH. It is expected that the results can provide an assessment of the performance improvement of the flexible humidity sensor and a reference for the research and development of intelligent wearable devices.

## 1. Introduction

Humidity sensors are widely used in smart wearables, environmental monitoring, healthcare, and food. With the rapid development of the smart wearable industry, the development of flexible humidity sensors with smaller feature sizes, lower costs, and better performance on flexible substrates has attracted much attention [1]. The electrode circuit with good conductivity and high precision is the crucial step in fabricating a flexible humidity sensor. Laser Direct Writing (LDW) technology is very suitable for sensor electrode circuits patterned on flexible substrates because of its characteristics of low sintering temperature, short processing cycle, high efficiency, high precision, large-area processing, a wide range of materials, and robust designability [2,3].

The choice of a flexible substrate and conductive ink is critical in the manufacturing of flexible sensor electrodes by LDW technology. According to the characteristics of the ink absorption spectrum, a laser can be selectively absorbed or transmitted by ink, so the appropriate laser wavelength selection is necessary, which can effectively prevent the damage to the flexible substrate caused by the direct absorption laser. Polyethylene terephthalate (PET) [4], polyethylene (PEN), polyimide (PI), polydimethylsiloxane (PDMS) [5], and paper [6] are usually used as flexible substrates. Among them, the PI with excellent mechanical properties, high-temperature resistance, corrosion resistance, obvious anisotropy formed in the manufacturing process due to the preferred orientation of the polymer chain, and other characteristics is regarded as an ideal flexible substrate material for the manufacturing of flexible electronic equipment [7,8]. Conductive ink is the critical material in the LDW manufacturing of a high-performance flexible circuit. The ink used in LDW is mainly the nanoparticles of metal or nonmetal with low cost. The nano metal oxide inks in non-metallic ink include nano NiO [9], nano CuO [10,11], nano Cu_2_O [12], and so on. Among them, the nano CuO ink, which can be reduced and sintered into a Cu electrode pattern by laser in the preparation of a flexible circuit [13], is a focus researched ink. Some research work has been carried out on the practice of Cu electrodes by laser reduction of nano CuO ink on flexible substrate PI. Kang et al. successfully fabricated a copper electrode with a thickness of about 10 μm on flexible substrate PI using nano CuO ink, which was low cost and stable in air, and they analyzed the photothermal and photochemical reactions after a plus and continuous laser irradiated the ink [14], which provided a solution for the manufacture of the flexible circuit. The choice of nano CuO ink, which possesses the advantages of nano Cu ink and solves the problem that Cu is easy to oxidize in air, is one of the solutions to the difficulty that metal ink oxidized quickly in flexible circuit preparation. Of course, metal or nonmetal oxide may be reduced inadequately or oxidated again after reduction, which can lead to the poor conductivity of the electrode circuits prepared by laser reduction. Hence, the laser reduction technologies of metal or nonmetal oxide ink still need to be explored for fabricating the conductive circuit with excellent electric and mechanical properties.

The electrode structure is also an essential factor affecting the sensor’s performance. The interdigital electrode is a widely used electrode structure and includes three primary parameters: interdigital width, length, and thickness. The optimization electrode structure with excellent performance, such as precision and sensitivity, can be found by simulation software to simulate and analyze the electric field [15], capacitance, [16] impedance [17], etc. generated by different electrode structures. Vakilian et al. [18] obtained the optimum size of interdigital electrode for biosensors by COMSOL software to analyze the capacitance value of the sensor influenced by different electrode structural parameters. The simulation results showed that the total capacitance of the electrode with the interdigital length of 100 μm increased by 50% when the interdigital width increased from 10 μm to 25 μm, the capacitance decreased when the interdigital distance fell from 25 μm to 10 μm, and the interdigital thickness had no significant effect on the interfinger capacitance, which proved that the optimum purpose could be attained by adjusting the interdigital width and distance to change the electrode characteristics. Although a lot of work has been completed on interdigital electrodes, the influence of electrode structure on the performance of LDW flexible humidity sensors has not been reported. In this study, CuO was reduced by LDW on a PI flexible substrate to produce Cu electrodes with various structures. The work is expected to provide ideas for the performance improvement of the flexible humidity sensors and a reference for the research and development of intelligent wearable devices.

## 2. Materials and Methods

### 2.1. Preparation of Nano CuO Ink

Nano CuO ink was obtained by mixing 60 wt% CuO (40 nm, Shanghai Macklin Biochemical Co., Ltd., Shanghai, China), 13 wt% polyethylene pyrrolidone (PVP, relative molecular weight of 10,000, K13–18, Shanghai Macklin Biochemical Co., Ltd., Shanghai, China), and 27 wt% ethylene glycol (HOCH2CH2OH, Tianjin Best Chemical Co., Ltd., Tianjin, China). To obtain a suitable concentration of ink for spin coating, the ink was diluted with DI water, mixed with magnetic stirring for 70 min, and treated by ultrasonic for 5 h.

### 2.2. Preparation of Humidity Sensor and Characterization

**Figure 1:**
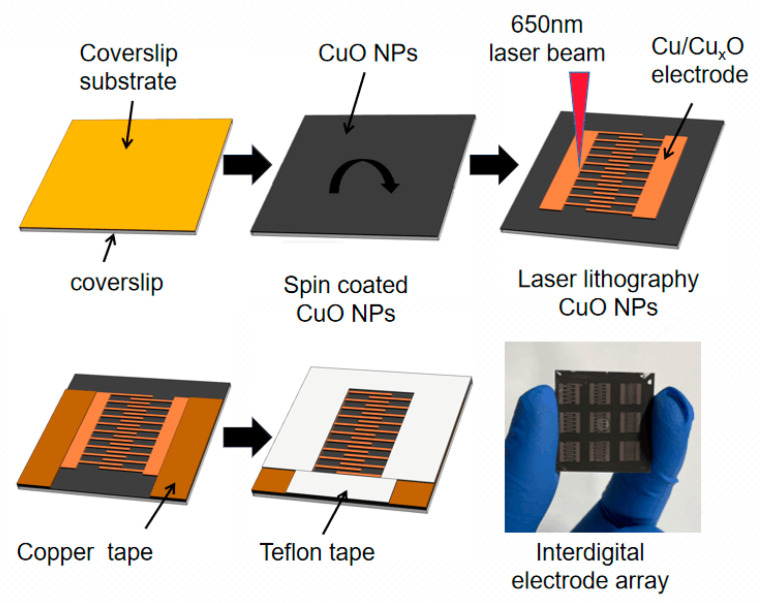
Schematic diagram of humidity sensor manufacturing process.

Commercial polyimide (PI) tape (Dongguan Shixin Packaging Materials Co., Ltd., Guangzhou, China) with a thickness of 80 μm was pasted on a cover glass with a thickness of 0.15 mm. Then, the nano CuO ink was dropped to the PI film to rotate at a speed of 1000 rpm/min for 30 s and dry for 1 h in air. The LDW device, which was equipped with a continuous-wave laser diode with a wavelength of 650 nm (power 100 mW, Philips E143838) and the direct current regulated power supply, was used to irradiate the ink. Electrodes printed by LDW were connected to copper tape as electrical test leads, and the sensor was encapsulated with Teflon tape. Figure 1 shows the preparation process of the humidity sensor.

The surface characteristics and element distribution of the sensor were examined by a scanning electron microscope (SEM) (MIRA3, TESCAN, Czech Republic) with an energy dispersive spectroscopy (EDS). The specimens were coated with gold before observation, and the operating voltage was 7.0 kV. The crystal structure of the ink material was investigated by X-ray diffraction (XRD) (XD-2, Puxitongyun, Beijing, China) equipped with Cu Kα radiation (λ = 0.1540 nm), operating voltage 40 kV, and current 30 mA.

### 2.3. Structure Design of Humidity Sensor

**Figure 2:**
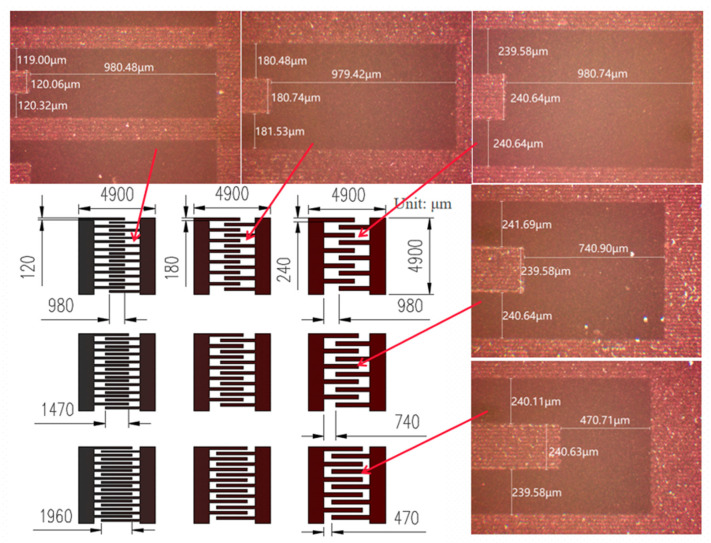
Design of electrode array and local optical micrograph.

To investigate the influence of a humidity sensor structure on its performance, the nine humidity sensors with various interdigital electrodes, whose interdigital widths respectively were 120 μm, 180 μm, and 240 μm and interdigital lengths were 1960 μm, 2210 μm, and 2430 μm, were designed based on 20 μm single lines. Figure 2 shows the designs and local optical micrographs of these interdigital electrodes. For comparison purposes, the dimensions of all sensors are 4900 μm × 4900 μm with the interdigital electrodes of equal width and spacing. As shown in Figure 2, the printing error accuracy of these interdigital electrodes is within ±1 μm, the line size is uniform, the electrode corner is flat, the interdigital width does not increase, and the electrode spacing does not decrease due to the energy accumulation at the edge, which is very critical in the manufacture of high-precision equipment or devices. Li et al. [19] prepared a LSG/GO humidity sensor by using a low power DVD driver. SEM results showed that the electrode with the width of 0.33 mm and the gap size of 0.33 mm was different from the original design. In the study, the error precision between the design size and the actual size of the interdigital electrodes is ±1 μm, which exceeds our expectation.

### 2.4. Performance Test of the Humidity Sensor

**Figure 3:**
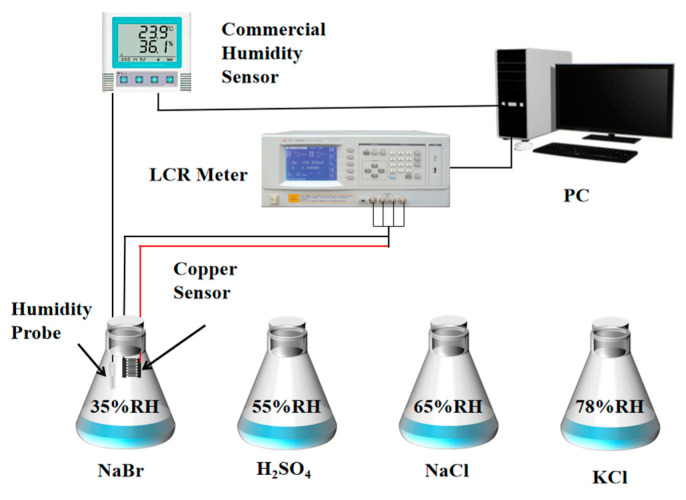
Schematic diagram of humidity sensing experimental device.

Four saturated salt solutions having specific relative humidity (RH), which were, respectively, sodium bromide (35% RH), sulfuric acid (55% RH), sodium chloride (65% RH), and potassium chloride (78% RH), were prepared as the performance test environment of the humidity sensor in the ambience (20 ℃, 20% RH). Figure 3 shows the testing device for humidity sensing. The sensor resistances were determined by an LCR tester (TH2828A, Changzhou Tonghui Electronics Co., Ltd., Changzhou, China) with a frequency of 1 kHz and a recording interval of 1 s. The test data were recorded by computer in real-time in various humidity environments. In the test, a commercial hygrograph (COS-03, Shandong Renke Control Technology Co., Ltd., Jinan, China) was used as a reference to record the changes in environmental humidity in real-time at an interval of 5 s. The sensor copper tape was connected to the LCR tester by a four-terminal fixture (TH26011B, Shandong Renke Control Technology Co., Ltd., Jinan, China) with shielded twisted pair.

The sensor’s sensitivity S is defined as follows [20]:

(1) $$S=(R_{0}-R)/\Delta \mathrm{RH}$$

where *S* is the sensitivity, *R*_0_ and *R* are the sensor’s resistance in air and humidity environments, respectively, and Δ*RH* is the variation in RH. Response time refers to the necessary time in which the sensor’s resistance is changed from the air to 90% RH, and recovery time refers to the essential time for which the sensor’s resistance is changed from 90% RH to the air.

## 3. Results and Discussion

### 3.1. Morphology and Structure Characterization of Materials

**Figure 4:**
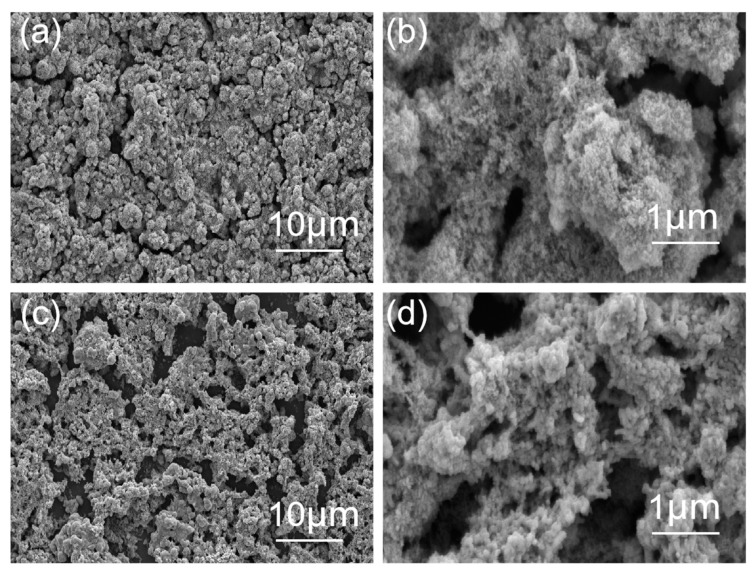
Morphologies of ink before/after laser irradiation: (**a**,**b**) before scanning; (**c**,**d**) after irradiation.

Figure 4 shows the SEM images of CuO ink before/after laser irradiation. As shown in Figure 4a,b, numerous cracks were left over from the evaporation of the solvent, and their sizes were about 1 μm width on the surface of the film before irradiation. After the film was irradiated, the cracks expanded from 1 μm to 3–5 μm, and the size of the CuO particle increased, as shown in Figure 4c,d. These cracks could be caused by the thermal expansion and contraction of the surface because of the uneven distribution of energy during laser irradiation [21]. The morphology of CuO is the agglomerated cauliflower in the form of an intensive stack before irradiation, and the morphology presents a coral-like structure for being fused and accumulated after laser irradiation [5].

**Figure 5:**
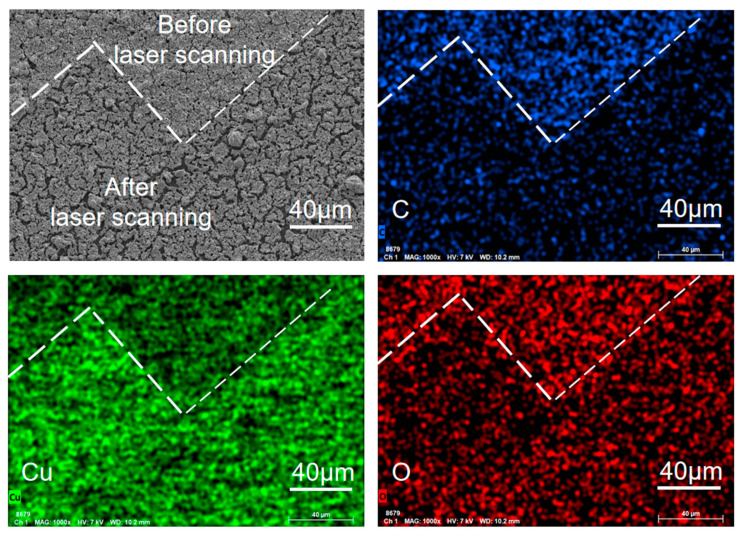
EDS image of the nano CuO ink surface after laser irradiation.

Figure 5 shows the energy spectrum analysis results of EDS on the ink surface. The distribution of C and O atoms are apparent differences on the surface before/after laser irradiating. There are few in the area irradiated by laser, which can be caused by the oxidization of C and O atoms and escaping in the form of gas during irradiation [5]. The content of Cu increases in the laser irradiation area, which indicates that CuO is reduced by carboxylic acid produced by the decomposition of PVP in ink under the high-temperature effect of the laser and is densely distributed in the non-fissure area.

**Figure 6:**
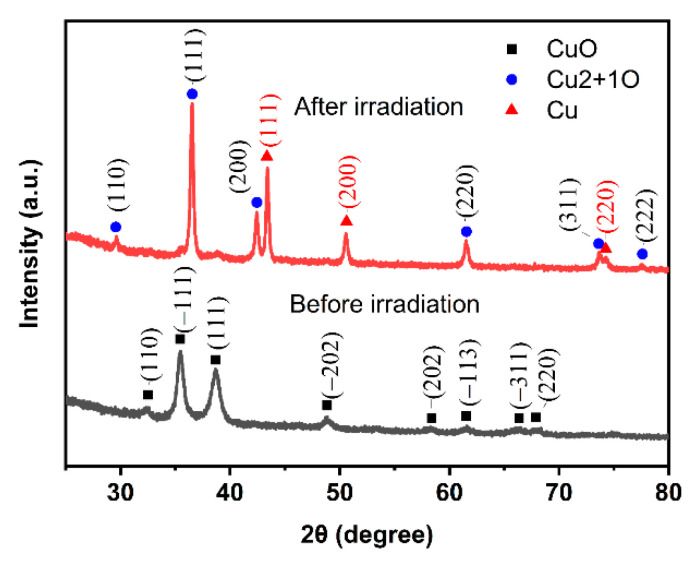
XRD patterns of the ink before and after laser irradiation.

Figure 6 shows the XRD patterns of the ink before and after laser irradiation. Before irradiation, the diffraction peaks at 2*θ* = 32.5°, 35.5°, 38.6°, 48.9°, 58.1°, 61.5°, 66.3°, and 68.0° correspond to (110), (−111), (111), (−202), (202), (−113), (−311), and (220) crystal planes of CuO monoclinic crystals, respectively (PDF#80-0076), and in the laser irradiation area, the diffraction peaks at 2*θ* = 43.4°, 50.5°, and 74.2° belong to the (111), (200), and (220) crystal planes of Cu (PDF-#04-0836), which indicates that CuO is reduced to Cu. The diffraction peaks at 2*θ* = 29.6°, 36.5°, 42.4°, 61.5°, 73.6°, and 77.5° are attributed to the (110), (111), (200), (220), (311), and (222) crystal planes of Cu2 + 1O, which is the excessive metal defect of Cu_2_O [22]. The XRD patterns confirm that the laser irradiation area is mainly composed of Cu and Cu2 + 1O. When the ink is irradiated by a laser, the CuO is heated, meanwhile, the glycol temperature is raised to near its boiling point and dehydrated to produce acetaldehyde at 160–200 ℃ [14]. Cu nanoparticles are made by reducing nano CuO with the acetaldehyde, and are condensed and sintered under photothermal action at the same time. The residual diacetyl and water, which are the secondary products generated in the photochemical reduction process, are evaporated in laser irradiation. Since the conversion from copper to cupric oxide requires a high temperature of 400 ℃, which cannot be reached in the laser sintering process, Cu2 + 1O is not reoxidized to copper oxide and is left as an intermediate phase [23].

### 3.2. Evaluation of Humidity Sensor Performance

**Figure 7:**
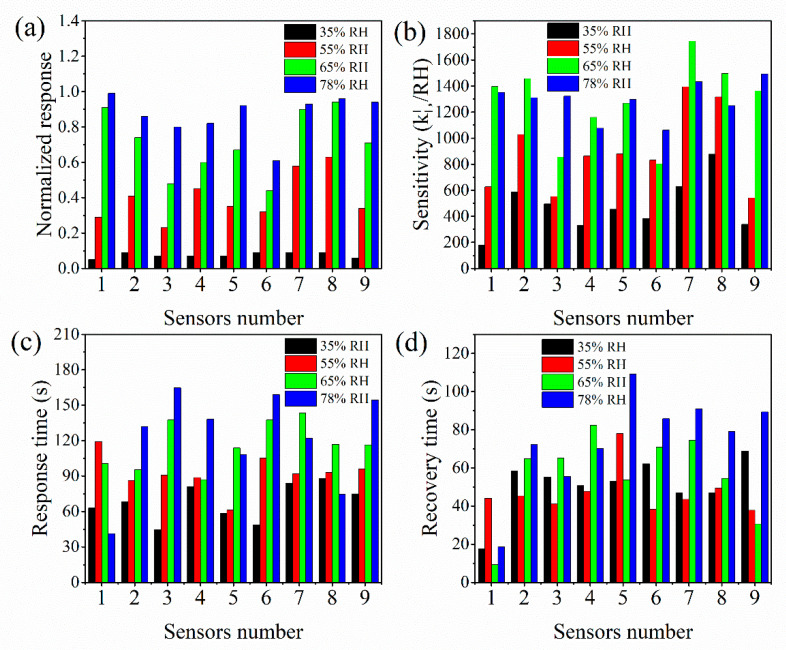
Sensing characteristics of humidity sensor in various humidities: (**a**) normalized response; (**b**) sensitivity; (**c**) response time; (**d**) recovery time.

The sensing characteristics of the prepared humidity sensor are evaluated and compared with commercial humidity sensors, including response recovery characteristics in the range of 35–78% RH. The humidity sensors with nine different electrode structures were numbered 1–9, and the electrodes were No. 1, 2, and 3 in the first row from left to right, respectively, and were No. 1, 4, and 7 in the first column from top to bottom in Figure 2. Figure 7 shows the normalized response of the resistance ((*R*_0_ − *R*)/*R*_0_), sensitivity, and response/recovery time of the sensors when relative humidity changes. The sensors show an excellent humidity response characteristic, and the normalized response value of the sensor increases with the increase in RH, as shown in Figure 7a. The normalized response value is less affected by the sensor structure in the low RH and is significantly affected in the high RH environment. Under 55%, 65%, and 78% RH, the normalized response values of No. 3, 6, and 9 sensors are smaller than other sensors in the same interdigital length, but with the interdigital width decrease, the normalized response values increase and reach saturation in 65% RH. The sensitivity of sensor 1 with a length of 1960 μm and a width of 120 μm is 180.2 KΩ/%RH and is lowest at 35% RH, and the sensitivity of sensor 7 with a length of 2430 μm and a width of 180 μm is 1744 kΩ/%RH and is highest at 65% RH, as shown in Figure 7b. The response time of sensors is 40–170 s in the 35–78% RH, and the response time of sensors 2, 3, 6, and 9 increases as the RH increases, as shown in Figure 7c. The recovery time of sensors is between 9 s and 110 s and irregular, except for sensor 8, as shown in Figure 7d.

**Figure 8:**
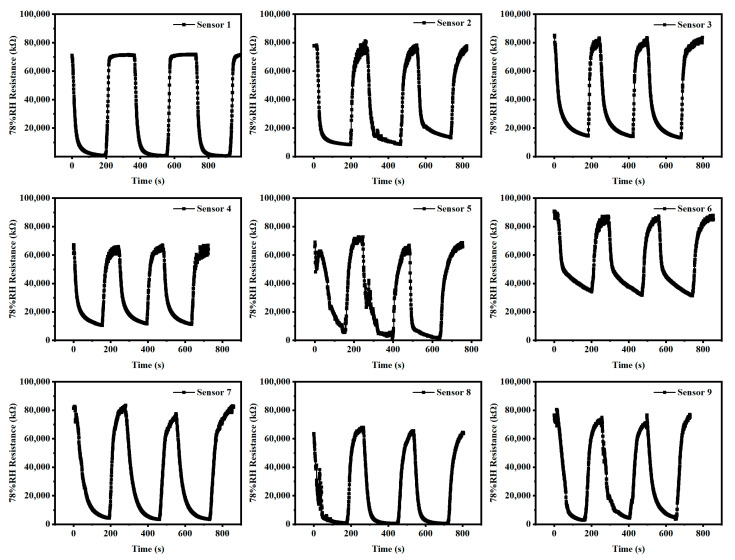
Resistance variation of nine humidity sensors with different electrodes in 78% RH.

Reusability is also an important index to evaluate the performance of humidity sensors. Figure 8 shows the resistances and recovery times of the reused humidity sensors with nine different electrode structures under 78% RH. The single response of these is about 250 s. The nine humidity sensors with varying electrode structures all offer excellent reusability in multiple cycle tests and maintain a high resistance response. That is, the sensor’s resistance can maintain up to 95% of the initial resistance from high RH to low.

**Figure 9:**
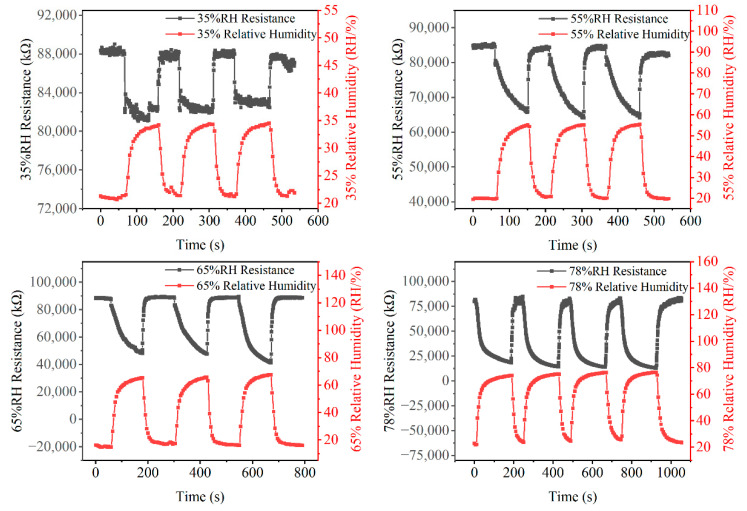
Comparison of humidity response between copper humidity sensor and commercial humidity sensor under various RH environments.

As an example, under 35%, 55%, 65%, and 78% RH, the resistance response of the No. 3 humidity sensor, because the response of all sensors is similar, and the testing data of the commercial humidity sensor are shown in Figure 9. The resistance response of the No. 3 humidity sensor is stable under various RH conditions. The resistance response changed by 6%, 28%, 49%, and 84%, respectively, from ambient humidity to 35%, 55%, 65%, and 78% RH.

Water molecules are adsorbed on the surface of the electrode, forming a thin coating of water when the sensor works. The performance of the humidity sensor can be affected by the adsorbance of water molecules, and the adsorption efficiency of water molecules largely depends on the structure of the interdigital electrode. Under low RH, the sensitivity of all the sensors is less affected by the electrode structure, which indicates that the adsorption efficiency of water molecules is similar to the copper-based humidity sensors. However, the adsorption efficiency of the sensor with a longer interdigital length is more robust under high RH.

Copper, which has a low price compared with other precious metals, such as gold and silver, and has good electrical conductivity, is considered as an excellent material for preparing a conductive structure. Cupric oxide and cuprous oxide, as two oxides of copper, are widely used in sensing systems for their excellent photoelectric and gas responsiveness [24]. Layered or porous nanostructures, which can provide more adsorption sites for water molecules because of higher specific surface area, are the best choice as sensing structures [25]. Of course, a denser structure is also needed to keep electrons moving as conductors. It was reported that CuO belongs to a p-type semiconductor, while Cu^0^ is preferable to incorporate into Cu_2_O lattices as a dopant in an acidic medium to form n-type Cu_2_O [26]. When these two semiconductors are in contact, the offset of work functions could create a space charge region through the diffusion of carriers with a built-in potential, which induces an electric field oriented toward the p-side. Peng et al. [10] printed the snake-like electrode with an adjustable ratio of Cu/Cu_x_O by LDW on a PC flexible substrate-covered Cu precursor film using the continuous wave of 808 nm. An integrated humidity sensor was prepared by combining a Cu_X_O-rich porous sensing structure with a Cu-rich conductive structure which showed high sensitivity to human respiration. In this study, nine humidity sensors with various electrode structures were designed to analyze the influence of electrode structures on humidity sensing. As a rapid machining process, LDW has been used to manufacture conducting circuits [27] and sensing material [28] in the past few years. In the laser patterning of the copper structure, the evaporation of the solvent in the precursor ink can form a porous structure during laser irradiation, which is beneficial for improving its sensitivity. Using a low power laser-induced reduction sintering technology [29], the reduction in CuO nanoparticles was achieved by photothermochemical reduction and agglomeration; therefore, the porous composite electrode of Cu and Cu2 + 1O, which can be used for humidity or photodetectors, was obtained by one-step reduction in CuO nanoparticles on flexible PI film [30].

## 4. Conclusions

The Cu and Cu2 + 1O electrodes were formed by one-step reduction in the nano CuO ink on a flexible PI substrate using LDW and were utilized as a humidity sensor. The results show that the narrowest interdigital width of the sensor is 120 μm, the most limited single line width is 20 μm, and the printing accuracy is ±1 μm. Nine humidity sensors with different electrode structures were prepared and the variation in their resistance was analyzed to be 16–78% RH. The sensitivity of the copper-based humidity sensor is hardly affected by the electrode structure under low RH, but is significantly affected under high RH. All sensors show excellent humidity sensing characteristics. The sensitivity of the sensor with a length of 1960 μm and a width of 120 μm is 180.2 KΩ/%RH and is the lowest under 35% RH, and the sensor with a length of 2430 μm and a width of 180 μm is 1744 kΩ/%RH and is the highest under 65% RH.

## Data Availability

The data presented in this study are available on request from the corresponding author.
